# Flower transcriptional response to long term hot and cold environments in *Antirrhinum majus*

**DOI:** 10.3389/fpls.2023.1120183

**Published:** 2023-01-27

**Authors:** Raquel Alcantud, Julia Weiss, Marta I. Terry, Nuria Bernabé, Fuensanta Verdú-Navarro, Jesualdo Tomás Fernández-Breis, Marcos Egea-Cortines

**Affiliations:** ^1^ Genética Molecular, Instituto de Biotecnología Vegetal, Edificio I+D+I, Plaza del Hospital s/n, Universidad Politécnica de Cartagena, Cartagena, Spain; ^2^ R&D Department, Bionet Engineering, Av/Azul, Parque Tecnológico Fuente Álamo, Murcia, Spain; ^3^ Department of Informatics and Systems, Campus de Espinardo, Universidad de Murcia, Instituto Murciano de Investigaciones Biomédicas (IMIB)-Arrixaca, Murcia, Spain

**Keywords:** cold stress, heat stress, adaptation, transcriptome, flower development, ribosomal genes, floral scent, phenylpropanoid metabolism

## Abstract

Short term experiments have identified heat shock and cold response elements in many biological systems. However, the effect of long-term low or high temperatures is not well documented. To address this gap, we grew *Antirrhinum majus* plants from two-weeks old until maturity under control (normal) (22/16°C), cold (15/5°C), and hot (30/23°C) conditions for a period of two years. Flower size, petal anthocyanin content and pollen viability obtained higher values in cold conditions, decreasing in middle and high temperatures. Leaf chlorophyll content was higher in cold conditions and stable in control and hot temperatures, while pedicel length increased under hot conditions. The control conditions were optimal for scent emission and seed production. Scent complexity was low in cold temperatures. The transcriptomic analysis of mature flowers, followed by gene enrichment analysis and CNET plot visualization, showed two groups of genes. One group comprised genes controlling the affected traits, and a second group appeared as long-term adaptation to non-optimal temperatures. These included hypoxia, unsaturated fatty acid metabolism, ribosomal proteins, carboxylic acid, sugar and organic ion transport, or protein folding. We found a differential expression of floral organ identity functions, supporting the flower size data. Pollinator-related traits such as scent and color followed opposite trends, indicating an equilibrium for rendering the organs for pollination attractive under changing climate conditions. Prolonged heat or cold cause structural adaptations in protein synthesis and folding, membrane composition, and transport. Thus, adaptations to cope with non-optimal temperatures occur in basic cellular processes.

## 1 Introduction

The response of organisms to high and low temperatures field of study has very solid knowledge based on short-term lab and field experiments. Many laboratory experiments have been performed with short exposures to changing temperatures, from minutes to hours, and up to several days. These experimental approaches, beginning in the mid-seventies, have shown that rapid responses to heat are a universal feature of bacteria, archeobacteria, fungi, plants, and animals. This rapid response to increased temperature raised the concept of heat shock, which coined the name of a complete set of genes involved in response to high temperatures, known as heat shock genes, which code for heat shock proteins (Lindquist and Craig, 1988). Experiments using cold stress in plants have identified the so-called cold acclimation pathways, activated by short exposures to low temperatures. After an initial period of heat or cold, plants adapt to non-optimal temperatures (Thomashow, 1998). However, climate change conditions, represented as hot or cold periods lasting longer than a few days, are poorly represented by such short-term experiments that explore the early players in adaptation processes, but not the long-term effect on biological systems.

Plant vegetative growth is coupled to reproductive development and success, and flower development is a highly conserved process controlled by flower organ identity genes (Egea-Cortines et al., 1999; Theissen and Saedler, 2001). A high expression of floral organ identity genes is required to achieve completely functional flowers in terms of size, color and scent emission (Manchado-Rojo et al., 2012). These characters are known as floral traits (Ramos and Schiestl, 2019). Flower color is one of the main floral traits used by pollinators to locate flowers. Aside from pigment concentration within the petal cells, petal color appearance relies on light reflectance by conical cells typical of the petal surface (Dyer et al., 2006; Glover, 2011; Brockington et al., 2013).

Together with color, floral volatiles function both as flower attractants to pollinators, as well as deterrents of parasites (Muhlemann et al., 2006). Floral volatile synthesis and emission is coordinated by several factors. These include flower age, pollination status, circadian regulation, and environmental conditions (Schuurink et al., 2006; Cna’ani et al., 2014; Weiss et al., 2016a).

The orchestration of a response against non-optimal temperatures occurs in several steps. The first genes identified as heat response genes code for the so-called heat shock proteins (HSP), and share the molecular function of dealing with protein misfolding and aggregation (Wang et al., 2004). As previously mentioned, short heat treatments lasting minutes in Arabidopsis, have an important impact on plant survival, thus coining the concept of adaptation to heat. The down regulation of HSP101 by interfering RNA decreases heat adaptation, whereas its overexpression enhances performance at high temperatures (Queitsch et al., 2000). The fast activation of heat responsive genes is downstream of several transcription factors such as HsfA2, DREB2A or bZIP28 (Sakuma et al., 2006; Schramm et al., 2006). Heat and cold stress share some signaling and transcriptional pathways, yet partially diverge in the output, as they activate differing gene networks. Cold responses occur *via* DREB2 and other transcription factors such as CBFs (Cook et al., 2004; Alonso-Blanco et al., 2005; Maruyama et al., 2009). The studies mentioned above share short periods of exposure to high or low temperatures, with the period of time under stress varying between one hour to a maximum of four days (Sakuma et al., 2006; Schramm et al., 2006; Gao et al., 2008; Gutha and Reddy, 2008; Maruyama et al., 2009; Bihani et al., 2011). Studies of cold acclimation usually comprise a period of one to two weeks at a mild temperature, followed by a period of freezing temperatures of up to six hours (Cook et al., 2004; Alonso-Blanco et al., 2005).

In the present study, we performed a long-term experiment using snapdragon as a model, as it is a semi-perennial ornamental plant (Stubbe, 1966). The aim of the experiments described was to identify the impact of long-term, non-optimal temperature, i.e. mild heat and cold in flower development. We discuss our work considering the current knowledge in the field of adaptation and survival under long-term non-optimal temperatures.

## 2 Materials and methods

### 2.1 Plant material and growth conditions

We used seeds of *Antirrhinum majus* inbred line 165E (Schwarz-Sommer et al., 2010). The seeds were germinated on fine vermiculite. The seedlings were transplanted to Nursery Plastic Pots (650 ml volume) after two weeks. During the entire treatment period, plants were watered as required. Plants were kept in Sanyo MRL350 growth chambers with day/night temperatures of 22/16 °C at a regime of 16 h fluorescent light at a photosynthetically active photon flux density of 250 μE s^−1^ m^−2^, and 8 h darkness.

Traditionally, Antirrhinum plants have been grown in greenhouses with temperatures ranging between 10°C at night and 28°C during the day. However, temperature effects have not been recorded for this plant. We established the initial conditions to grow *Antirrhinum* plants under long-term high and low temperatures. Traditionally, Antirrhinum plants have been grown in greenhouses with temperatures ranging between 10°C at night and 28°C during the day. Plants were grown for two weeks at a thermoperiod of 22/16**°**C and a photoperiod of 16/8 light/dark corresponding to long days. These long day conditions induce flowering in *Antirrhinum* (Bradley et al., 1996). After two weeks, the plants were transferred to their final temperature growing regimes for the rest of their development. Initially, the high temperatures were set at 34/28°C, but the plants did not survive, and we could not obtain flowers for further work. Thus, we decreased the temperature to 30/23°C where most plants survived producing a few flowers. Plants grown in the cold were kept at 15/5°C, and they developed slowly. The plants were kept under control, heat, or cold conditions for a minimum of four months, with some of them for over two years, as flowering was strongly delayed (data not shown). During this period, we sampled flowers from the different treatments.

**Figure 1:**
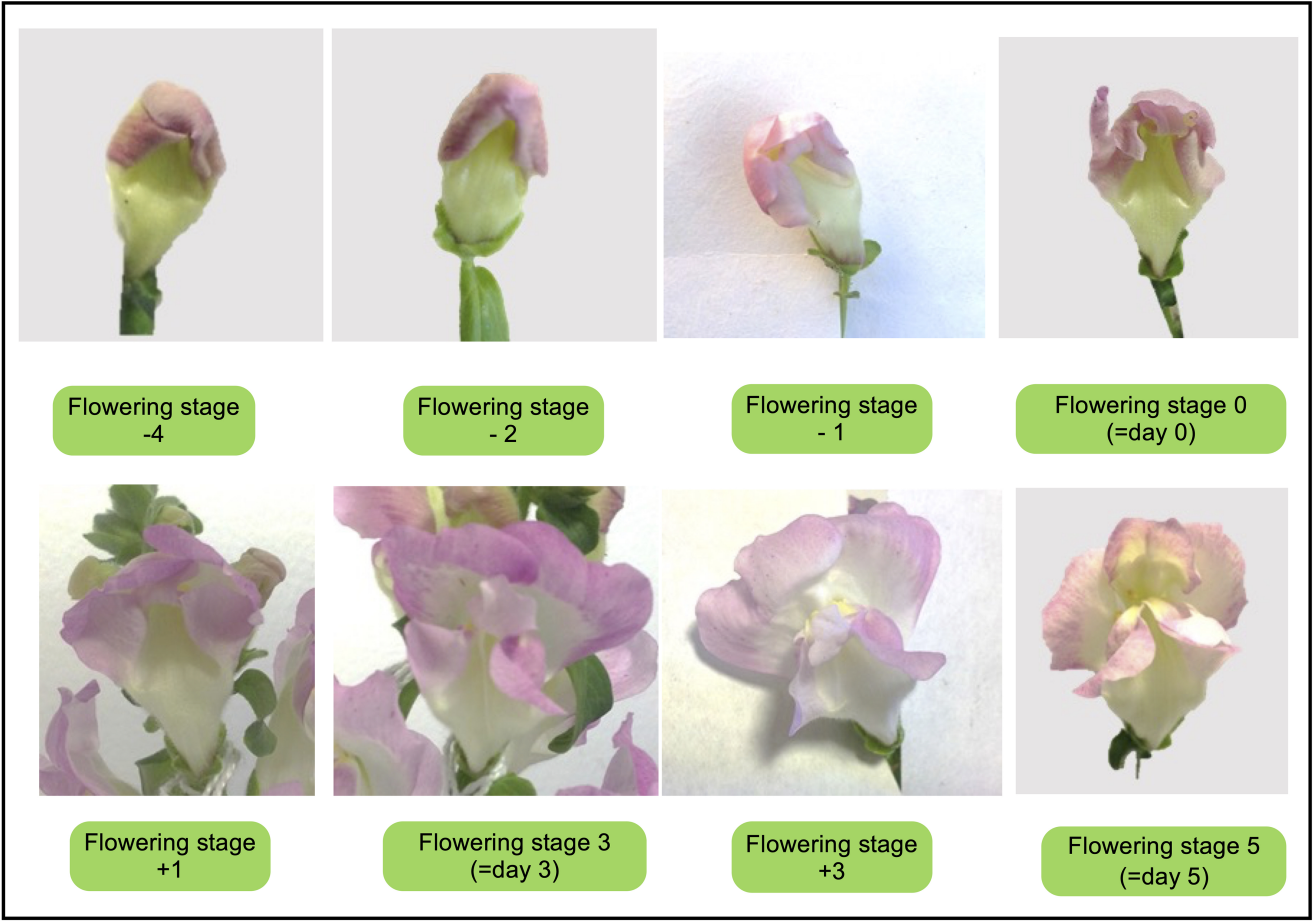
Flowering stages of *Antirrhinum majus* line 165E. Flowering stage range from -IV (before flower opening) until stage V (day 5 after flower opening).

Flower developmental stages were categorized as shown in **Figure 1**: Flowering stage 0 coincided with day 0 or anthesis. The sampling of flowers was performed on days 0, 3, and 5 days after anthesis (DAA). Flower organ parameters were selected as described previously (Weiss et al., 2016b) and were measured in flowers on day 3 (**Figure 1**).

### 2.2 Pigment measurements

We extracted total anthocyanins in flowers from 0.1 g of petal tissue at day 3 after anthesis using a methanol-HCl method, and spectrophotometric absorption measurement at 530 and 657 nm (Zhang et al., 2019), using a UV-1600 spectrophotometer (SHIMADZU, Kyoto, Japan). The extraction was performed on nine flowers from three different plants per temperature regime. Chlorophyll was measured in apical, median, and basal leaves with a CM-500 Chlorophyll Meter, which determines the relative chlorophyll content by measuring the light penetration coefficient in a 2-wavelength range corresponding to red light (660nm) and IR light (850 nm).

### 2.3 Scanning electron microscopy analysis

We sampled adaxial (upper) petal regions encompassing areas 6, 7 and 8 as described previously (Delgado-Benarroch et al., 2009b) with a scalpel blade. Petal sections of approximately 0.75 cm^2^ were prepared for scanning electron microscopy. The cell size of flowers from different temperature regimes were measured based on the cell area of 40 cells per preparation using the program ImageJ (https://imagej.nih.gov/ij).

### 2.4 VOC analysis

We sampled flowers at day 0, day 3 and day 5 from the control, low and high temperature treatments. This resulted in nine experimental groups.

We collected volatiles with polyvinyl siloxane coated bars (Twister^®^ Gerstel), as previously described (Ruiz-Hernández et al., 2018). Briefly, every snapdragon flower was cut, weighted, and placed in a glass beaker with 4 mL of a 4% sugar solution. We attached a Twister to each beaker, after which it was placed in a glass desiccator. We removed the Twister after 24 hours. The control consisted in a beaker with the sugar solution without snapdragon flowers, which was also placed in a desiccator. Once the Twisters were removed, they were stored at 4°C until further analysis. For each flower stage and temperature conditions, we sampled 8 flowers, except for the control group and 5 DAA, which consisted of 7 samples. In total, we collected volatiles from 71 snapdragon flowers.

The VOCs adsorbed by the Twisters were separated using a GC-MS HP-6890N coupled to a 5975 mass-spectrometer (Agilent Technologies, Palo Alto, CA, USA), combined with a TDU and cooling injector system (CIS4) (Gerstel).

The Twisters were desorbed by heating, starting from an initial temperature of 40°C to 250°C, at a ramping speed of 100°C min^−1^, with 5 min hold time on a splitless mode. The desorbed compounds were captured in a cool trap at − 100°C. The process was automated by using an MPS2XL multipurpose sampler (Gerstel).

The chromatograms were obtained with a HP5MS-UI column (Agilent Technologies), with helium as the gas carrier, in constant pressure mode and a 1:50 split ratio. The initial temperature was 50°C, increasing at a ratio of 5°C min^−1^ until 70°C, after which the temperature was held for one min. In the following step, the temperature was increased to 240°C at a rate of 10°C min^−1^, and held for 15 min.

The mass spectrometer operated at a 70 eV ionization voltage. The source and quadrupole temperatures were 230 and 150°C, respectively. The mass range was 30.0 to 450.0 uma at 4 scan/s. The MSD transfer line was maintained at 280°C.

We used the ChemStation software (version E.02.02 SP1, Agilent Technologies) to acquire chromatograms. The compounds were qualitatively identified by comparison with the Wiley10th-NIST11b mass spectral database (Agilent Technologies, Wilmington, DE, USA).

Once we integrated all the chromatograms, we used the R package gcProfileMakeR to obtain the scent profile (Perez-Sanz et al., 2021). We determined the VOCs that were present in all the samples of a given treatment. We will refer to this first profile as “constitutive scent profile”. Additionally, we evaluated the volatiles present in more than 70% of the samples. To obtain these profiles, we set the gcProfileMakeR parameters as follows: pFreqCuttof = 1 (constitutive profile or volatiles detected in all samples). In addition, we removed contaminating VOCs such as PDMS-derived volatiles as hexamethylcyclotrisiloxane, with the cas2rm function, and setting the minQuality to 80.

The volatile amounts, expressed as integrated peak area divided by flower fresh weigh, were compared among snapdragon groups and flower stages using Dunn test, implemented in the R package FSA.

### 2.5 Pollen viability analysis

Pollen viability was evaluated with pollen from seven to ten flowers at day 3 after flower opening, grown under standard conditions as well as high and low temperature regimes. The pollen was tested for the presence of cytoplasm by aniline blue staining with 0.1% aniline blue in 0.1N K_3_PO_4_ at pH 8.5 (Kho and Baër, 1968).

### 2.6 Capsule development and seed germinability

Flowers developing under the different temperature regimes were hand self-pollinated, and total seed weight and seed number of 10 capsules from 3 plants were recorded. From each capsule, we performed a germinability assay by placing 20 seeds on a filter paper moistened with distilled water in a petri dish, which was kept under darkness in a growth chamber at 22°C. The germinated seeds were counted after two weeks.

### 2.7 Statistical analysis

To test differences in flower parameters, chlorophyll, and anthocyanin content between the different temperature groups, we used the non-parametric Wilcoxon test, implemented in R. Their respective graphs were plotted by using the R package ggplot2 (Wickham, 2011, 2). A Principal Component Analysis (PCA) of the VOCs was performed using log-transformed data and expressed as a proportion of the total volatile amount (Tang et al., 2016).

### 2.8 Transcriptomic analysis

Total RNA was isolated from petal tissue using the NucleoSpin RNA plant kit (MACHEREY-NAGEL, https://www.mn-net.com/) which includes DNase. The quality and concentration of the RNA was determined spectrophotometrically with a NanoDrop ONE (Thermo-Fisher). First strand cDNA was synthesized using 500 ng of total RNA with Maxima kits (Thermo-Fisher), according to the user’s manual. Due to differences in flower size resulting from the treatments, we took five flowers per sample of cold, control and heat treatment for a total of 15 samples. We used five samples per treatment corresponding to five biological replicas to perform the transcriptomic analysis.

The quality and concentration of total RNA samples were analyzed before the experiment to ensure sufficient integrity and quantity. To achieve this, the RNA integrity number (RIN) of each sample was investigated. The Kapa Stranded mRNA Library Preparation Kit was used for the library construction of cDNA molecules. Furthermore, the generated DNA fragments were sequenced using the Illumina Hiseq 4000 platform with 150 bp paired-end sequencing reads (Stabvida, Portugal). We obtained a total of 992 million reads comprising 149 Gbp of DNA. The raw data can be downloaded from the European Nucleotide Archive (ENA) https://www.ebi.ac.uk/ena/browser/home (Study: PRJEB54068, Samples: ERS12336218-ERS12336232, Experiments: ERX9450224-ERX9450238, Runs: ERR9907396-ERR9907410)

HiSat2 (version 2.1.0) (Kim et al., 2019) was used to align the RNA-seq reads to the genome assembly version 2 of *Antirrhinum majus* available at http://bioinfo.sibs.ac.cn/Am (snapdragon.chr.IGDBV1.fasta). StringTie (Kovaka et al., 2019) (version 2.0) was used for assembling the transcripts and estimating abundances. We also used the gene annotation of this assembly of the genome (snapdragon_IGDBV1.chr.gene.gff). The NOISeq (Tarazona et al., 2011) R package (version 2.31.0) was used for the differential expression tests, accepting results with probability of differential expression ≥ 0.95. The data analysis and the graphical representations were done using an in-house R script.

The enrichment analysis and the CNET plots were performed by using the ClusterProfiler R package (version 3.16.1) (Yu et al., 2012), using 0.05 as thresholds for the p-value and q-value. An in-house R script was used for generating an annotation package for *Antirrhinum majus* compatible with ClusterProfiler. The GO annotation for biological processes and molecular functions for the genome assembly version 2 of *Antirrhinum majus* was obtained from http://bioinfo.sibs.ac.cn/Am. However, as the number of annotated genes was 20,820, we annotated further for a total of 26,109 genes (out of 37,234). The description of this additional annotation process and the resulting files are available at https://github.com/jesualdotomasfernandezbreis/snapdragon-annotation.

## 3 Results

### 3.1 Changes in flower size as a response to temperature are organ specific

**Figure 2:**
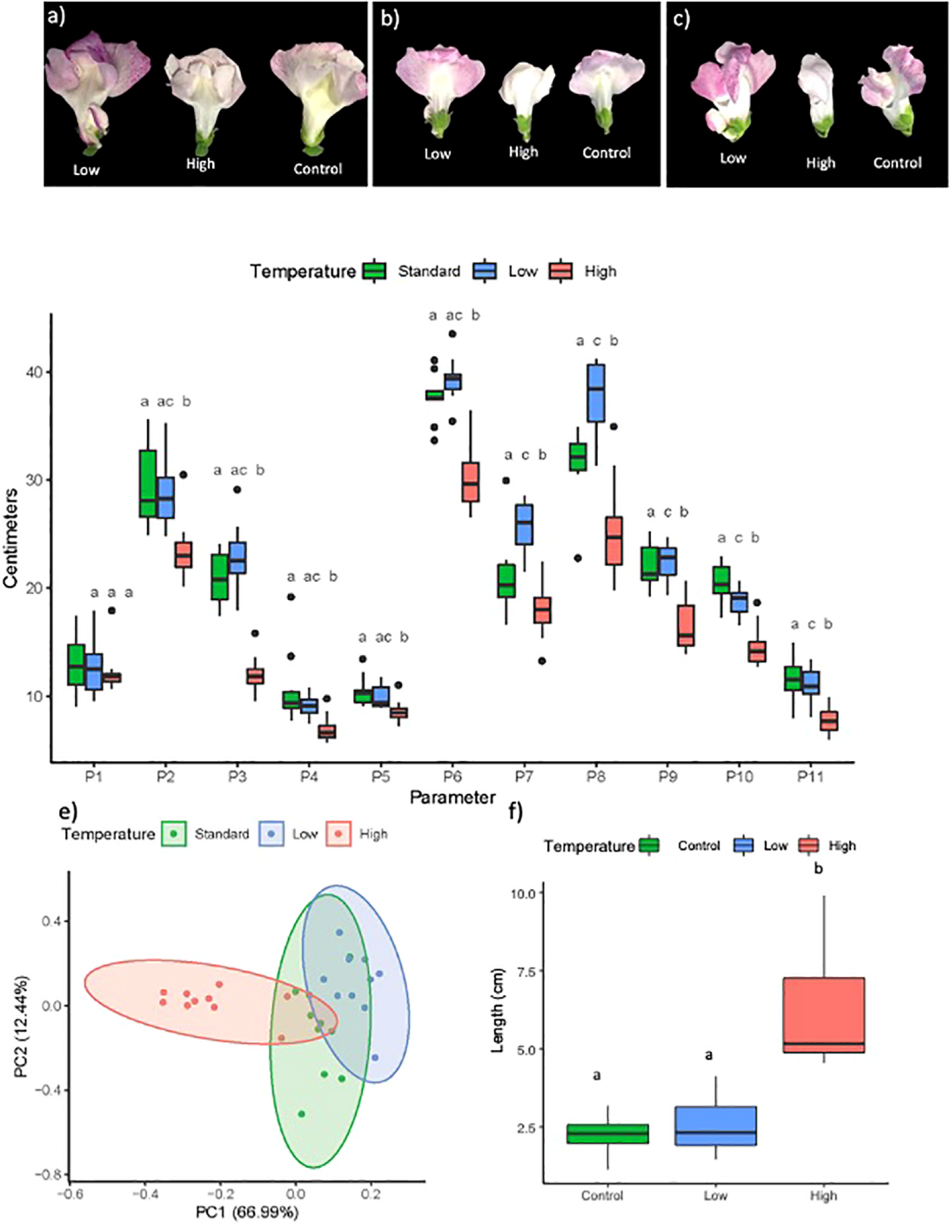
Images of **(A)** front, **(B)** back and **(C)** lateral view of flowers of *Antirrhinum majus* kept during inflorescence development at low, high and standard temperatures. **(D)** Flower parameters **(P)** under standard, high and low temperatures. P1: petal tube length: P2: lower petal length; P3: petal height: P4: sepal length; P5: tube width; P6: upper petal length: P7: lower petal expansion; P8: upper petal expansion: P9: stamen length: P10: gynoecium length: P11: pallate expansion. Different letters for each parameter indicate significant differences according to Fisher's F test or Wilcoxon test (see Supporting Information **Table S1**) (Sepal length and upper petal expansion). **(E)** PCA of floral weight and flower parameters. Each point represents one of the flowers analyzed. **(F)** Pedicel length (see Supporting Information **Table S2**).

Flower size was significantly affected by temperature. Flowers from plants grown under standard temperature conditions had an average weight of 255 mg. Development at cooler temperatures resulted in 15% heavier flowers, while higher temperatures produced 32% lighter flowers (**Figure 2**, **Table S1**). We measured eleven flower parameters: P1: petal tube length; P2: lower petal length; P3: petal height; P4: sepal length; P5: tube width; P6: upper petal length; P7: lower petal expansion; P8: upper petal expansion; P9: stamen length; P10: gynoecium length; P11: palate expansion. All flower parameters were significantly reduced at high temperatures as compared to control conditions, except for petal tube length (P1), which remained stable (**Figure 2D**, **Supplementary Table S2**). Lower temperatures resulted in significantly increased values of the lower and upper petal expansion (P7 and P8, **Figure 2D**). Gynoecium length (P10) was significantly reduced, while other parameters were not significantly different as compared to control conditions (**Figure 2D**).

To identify overall changes in flower parameters in plants under the different conditions, we performed a PCA analysis (**Figure 2E**). The first principal component explained 66.99% of the variance, corresponding to upper petal length, and the second principal component explained 12.44% of the variance the data, corresponding to sepal length. The data showed that the flower structure was similar under low temperatures as compared to control conditions, while heat drastically affected petal growth in most parameters. Pedicel length doubled in flowers grown under high temperature conditions, as compared to control conditions, while flowers from control and low temperature conditions had a similar pedicel length (**Figure 2F**, **Supplementary Table S3**).

**Figure 3:**
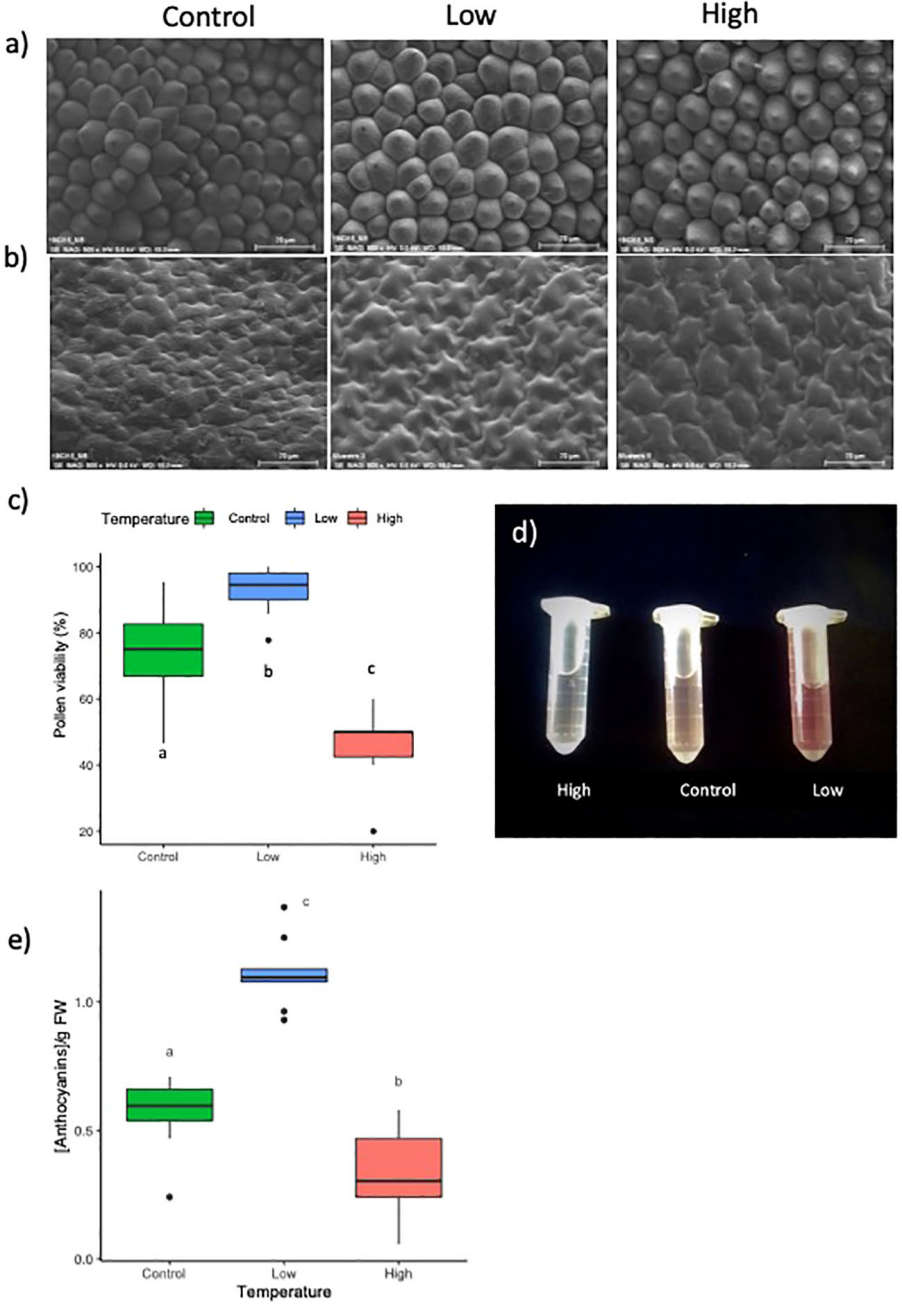
**(A)** From left to right, conical cells and **(B)** flat cells at control, low and, high temperatures. **(C)** Pollen viability at control (green) low (blue) and high temperature (red), expressed as percentage. **(D, E)** Total anthocyanin content in petals of *Antirrhinum majus* flowers grown under control, low, and high temperatures (see Supporting Information **Table S3**). Letters show significant differences between samples.

As petal size showed significant differences under different temperatures, we analyzed cell size by SEM. Cell area in the adaxial petal region, encompassing regions of conical and flat cells, were not significantly different between temperature regimes for either cell type (**Figures 3A, B**; **Table 1**). This indicates that the temperature effects on flower size were the result of changes in cell division that translated into larger or smaller flowers, while cell expansion was probably not affected. We did not observe changes in flower opening, a process requiring local cell expansion (Van Doorn and Kamdee, 2014; Sun et al., 2016).

**Table 1:** Cell area in flowers of *A.majus* under control, cold and hot conditions.

Temperature	Conical cells	Flat cells
Control	849.09 ± 131.89	1401.00 ± 144.38
Cold	878.04 ± 10.73	1397.53 ± 98.79
Hot	770.85 ± 106.52	1591.45 ± 177.25

Total number of cells per treatment n= 40. Values represent average area (μm^2^) and standard deviation. Statistical differences with T-test show no differences between cell types.

### 3.2 High and low temperatures significantly influence pollen performance and seed set

The different temperature regimes also affected the quality of the pollen (**Figure 3C**). While a higher temperature significantly reduced pollen viability from 74.5% under standard conditions to 45%, lower temperatures increased viability to 93%. We hand-pollinated autogamous *A. majus* flowers under the different temperature regimes to evaluate the possible effects on fertility, capsule development, and seed set. High and low temperatures did not affect seed germinability significantly. However, while the average percentage of seed germination was comparable between control and low temperatures, this factor increased 1.5 times at higher temperatures (**Table 2**). On the contrary, the number of seeds was significantly reduced to 46% at a low temperature and was also reduced by 16% at a high temperature, although this change was not significant. No significant differences were observed concerning seed weight. We can conclude that cold temperatures increased pollen germination, while heat has a strong negative effect. However, seed number appeared to be at an optimum at control and high temperatures, as cold treatments decreased seed numbers.

**Table 2:** Effect of temperature on seed germination and size.

Temperature	% Germination	N° of seeds/10 capsules	Seed weight (gr)
Standard	29a	1404.00 ± 59.34a	0.025 ± 0.029a
Cold	25a	755 ± 26,18b*	0.012 ± 0.003a
Hot	46b**	1175.53 ± 39.81c*	0.007 ± 0.002b*

The asterisks refer to p values of 0.05 (*) and 0.01 (**). Statistical differences with T-test are marked by letters.

### 3.3 High and low temperature regimes affect flower pigmentation but not petal cell structure

Temperature had a significant effect on petal pigmentation (**Figures 2A-C**; **Figure 3D**). As compared with the control temperature condition, cooler temperatures resulted in visually darker pink to red pigmentations. In contrast, higher temperatures produced flowers with a paler color, sometimes appearing completely white. Petal color appearance depends both on anthocyanin concentration and conical cell structure due to light reflectance (Noda et al., 1994). We analyzed total anthocyanin content and found that the apparent color change coincided with a significantly higher total anthocyanin content in petal tissue under cold temperatures, and a significantly lower total anthocyanin content in petal tissue under higher temperatures, as compared to standard conditions. (**Figure 3E**, **Supplementary Table S4**). As conical cells that form the petal epidermis play a key role in light reflectance, we studied the floral epidermal structure by SEM. We did not observe any apparent difference in the structure of conical or flat cells in petals from the different treatments (**Figure 3A**).

We also analyzed the effect of temperature regime on leaf pigmentation (**Supplementary Figure S1**, **Table S5**). Just as the petal anthocyanins, leaves kept under lower temperatures showed a significantly higher relative chlorophyll content in apical, median, and basal leaves. However, under high temperatures, we did not observe significant differences in chlorophyll as compared to control leaves.

Altogether, we can conclude that low and high temperatures have opposite effects on flower anthocyanin concentrations, although the temperatures used did not interfere with conical or flat cell morphogenesis in petal epidermis. Chlorophyll concentrations are affected by cold but not by heat.

### 3.4 Temperature affects quantity and quality of volatile compounds

We analyzed the effect of temperature on floral scent emission. We observed significant differences in 1) total emission 2) changes in total emission during aging, and 3) changes in the VOC profile during aging.

**Figure 4:**
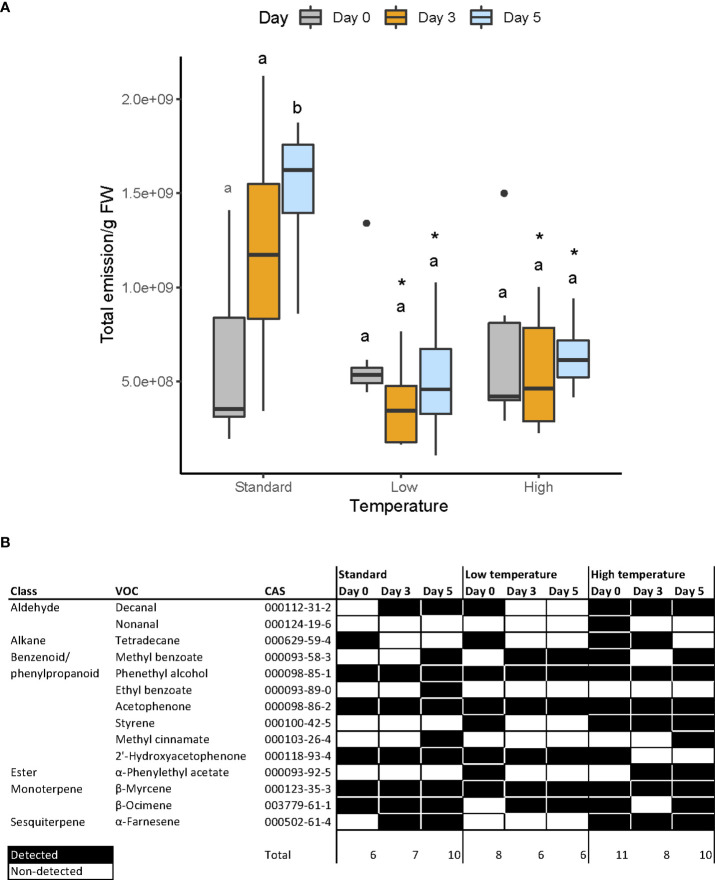
**(A)** Total floral scent emitted by flowers. Letters over bars indicate differences across each group (see Supporting Information, **Table S5**) whereas asterisks denote differences across flower stages (see Supporting Information, **Table S6**), **(B)** Constitutive scent profile of snapdragon flowers under three different conditions (standard, low and high temperatures) and at three development stages, expressed as days after anthesis. This profile comprises those volatiles that were present in all samples from each group. Black and white cells indicate constitutive and non-constitutive compounds, respectively.

Flowers grown under control conditions showed increased VOC emission after flower opening, which was significantly higher at day 5 as compared to day 0 or day 3 (**Figure 4A**). In contrast, flowers grown at low or high temperatures showed a stable VOC quantitative emission throughout development, and did not display increased VOC emission late in development (**Figure 4A**). When we compared the effect of temperature on total emission within treatments, we found that flowers grown under hot conditions emitted similar amounts as those grown under control conditions at 0 DAA (**Figure 4A**, **Supplementary Table S6**). However, emission was significantly lower at 3 and 5 DAA, 53% and 68% as compared to control flowers (**Figure 4A**, **Supplementary Table S7**). Under cold conditions, VOC emission again started at similar levels as control plants, but the reduction in emission was more pronounced. VOC emission was significantly lower, amounting to 25% and 29% at day 3, and 5 DAA, respectively, of the VOCs emitted under control conditions.

We analyzed the VOC profile of the different temperature regimes at days 0, 3 and 5. We identified a total of 111 different volatiles comprising alcohols, aldehydes, phenylpropanoids, fatty acid derivatives, and mono- and sesquiterpenes (**Supplementary Figure 2**). We defined the volatile profile into two overlapping datasets. The constitutive scent profile, i.e. the volatiles present in all flowers of a given treatment at a given age (**Figure 4B**), and the non-constitutive (**Supplementary Figure 2**), found in 70% of a given temperature and age group. The constitutive profile consisted in a set of 14 volatiles (**Figure 4B**). We found phenethyl alcohol, acetophenone, 2’-hydroxyacetophenone, and myrcene present in all flowers analyzed irrespective of age or temperature treatment. Thus, these VOCs can be considered the basic or core volatilome of this snapdragon line. We observed that the complexity, i.e. the number of detected volatiles, varied across temperature and flower stages. Plants grown under control conditions emitted a total of 11 constitutive compounds. The number of VOCs increased from six up to ten between day 0 and day 5. Under high temperature conditions, the profile was more complex, with the emission of a total of 13 different compounds. It started with 11 VOCS, and decreasing to eight and ten. In contrast, the VOC profile under cold conditions was the poorest, with a total emission of ten volatiles, starting with eight and decreasing to only six. When we analyzed the non-constitutive profile, it included 111 different compounds (**Supplementary Figure 2**). We observed that at DAA 0, the control group had the highest number of VOCs (52), decreasing progressively to 49 at day three and 31 at day five. The complexity of cold grown flowers was smaller, with 27 VOCs at day 0, 24 at day three and 16 at day five. Under hot conditions, the VOCs showed an overall higher complexity, starting with 44 at day 0, 51 at day three, and 45 at day five. Overall, cold-grown plants produced a subset of the complete volatilome found in normal conditions, while heat-grown plants maintained a somewhat more complex volatilome at day five.

In addition, some volatiles were not affected by temperature, and were emitted at the same flower stage. Thus, at 0 DAA, we identified 2’-hydroxyacetophenone and tetradecane. However, at 5 DAA, all snapdragon flowers emitted methyl benzoate and ocimene.

Some volatiles were detected in 70% of the samples of a given treatment and age (**Supplementary Figure 2**). This dataset showed that some VOCs were produced at control and high temperatures, but not at cold temperature. These included volatiles of different chemical types, indicating a general inhibitory effect of cold temperatures on floral scent complexity.

### 3.5 Transcriptomic signature of long-term growth under low and high temperature

We used petals at day 3-5 to obtain a comprehensive transcriptomic profile. We obtained a set of 2202 genes that were differentially expressed between the cold and control conditions, 3994 between hot and control conditions, and 5384 when we compared hot vs cold conditions.

We performed a Gene Enrichment Analysis followed by CNET plots depicting the GO terms and KEGG pathways. We found several gene networks confirming the temperature phenotypes observed in the control and treatment flowers. There were several categories in biological processes found in hot vs cold (**Supplementary Figure S3**). These included phenylpropanoid synthesis, sulfur compound metabolic process, response to toxic processes, and response to heat. A set of genes showed GO terms related to changes in unsaturated fatty acid metabolism, and a second set to hypoxia (see below). Cold showed two meaningful but unrelated CNET networks, one was pollination, including terms such as unidimensional cell growth or cell tip growth, and again, a second network with an increased number of genes involved in response to hypoxia. Additional related terms included response to oxygen levels or cellular response to decreased oxygen levels. When we analyzed hot vs cold, we found basically the sum of terms, plus additional ones including response to chitin, response to salicylic acid, or antibiotics.

Pollen viability followed a continuum from cold to heat conditions. Amongst the 345 genes enriched with the GO term pollen development, *Am06g26270* and *Am04g06020* are two MADS-box transcription factors orthologous to *AGL65* and *AGL66*. The AGL55 and AGL66 proteins interact *in vitro* and *in vivo* with the ARID‐HMG DNA‐binding protein AtHMGB15 (Xia et al., 2014). The ortholog of *AtHMG15*, *Am01g14540*, was also differentially expressed, and this transcriptional complex is required for pollen tip growth in Arabidopsis. Indeed, other genes such as *Am05g01300*, a WD40 transducin involved in pollen growth (Wang et al., 2008), *Am03g02130*, an SRK lectin of the S-locus (Goubet et al., 2012), several lectin receptors, and protein kinases, were differentially expressed when comparing hot vs cold. Additional genes involved in pollen development included *Am01g51790*, a calcium-dependent protein kinase known to play a role in cell polarity required for pollen growth in petunia (Yoon et al., 2006). Several Rho-encoding genes involved in microtubule growth, and *Am06g24120*, coding for an Armadillo protein, were differentially expressed. Armadillo proteins confine Rho to pollen tips, where they promote growth (Kulich et al., 2020). This indicates that the impact of temperature on pollen viability is the result of a major disruption of several independent pathways.

An important question we had was what components are found with GO terms related to stress under long-term temperature changes. The largest sets of biological process (BP) genes were related to response to toxic substances (322 genes), response to antibiotics (311 genes), hypoxia (210), and response to salicylic acid (SA) (188 genes). These four sets were largely overlapping. In fact, all the SA response genes were found within the response to antibiotics category and toxic substances. Furthermore, from the 245 transcripts related to heat, all of them except 24 were found within the toxic substance GO term. This indicates that long term non-optimal temperatures cause a steady transcriptional signature related to stress. We identified 245 transcripts with a heat enrichment term. Amongst them, we found several bona fide heat shock genes. These included 20 HSP20, 38 HSP70, four HSP90, 14 DNAJ type chaperones, and 13 FKBP-type peptidyl-prolyl cis-trans isomerases with chaperone activity. This revealed that the protein folding machinery was activated by heat at early stages and maintained throughout development.

While hypoxia occurs in plant roots under water logging (Huang and Johnson, 1995), it also appears to play a role in the maintenance of the stem cell niche in the shoot apical meristem (Weits et al., 2019). Several transcription factors found in the transcriptional network, which were activated during hypoxia, were differentially expressed. These included AP2 transcription factors such as ATRAV1-EDF4, a gene involved in response to hypoxia, activated by cold in a circadian fashion and transported between cells (Thieme et al., 2015), *ERF9*, *DREB2* (*Am01g12910*, *Am03g34650*, *Am01g54280*) and *DREB2A* (*Am07g22160*) (Mustroph et al., 2009). Additional AP2 members differentially expressed included *Am07g28230 and Am07g28230* (RAP2.2) *Am42820* corresponding to *DREB26* and *Am08680* (*ERF114*). A second set of genes included four WRKY genes, three with high homology to *WRKY70*, and one more closely related to *WRKY22*, a gene sharing a hairpin sequence acting as a thermo-switch (Chung et al., 2020). At least ten independent genes coding for alpha crystallin/HSP20 were enriched, coinciding with reports in a variety of plants (Lasanthi-Kudahettige et al., 2007; Ji et al., 2019). Amongst the enzymes responsive to hypoxia, we found an ortholog of ADH1 involved in alcohol metabolism (Loreti et al., 2005).

The molecular functions corresponding to the previous GO biological function terms included several independent gene networks that were enriched in heat vs control (**Supplementary Figure S4**; **Figure 6**). These included terms related to stress such as ribosome structure, glutathione transferase, unfolded protein binding, and others related to metabolic processes including unsaturated fatty acid and phenylpropanoid metabolism.

**Figure 5:**
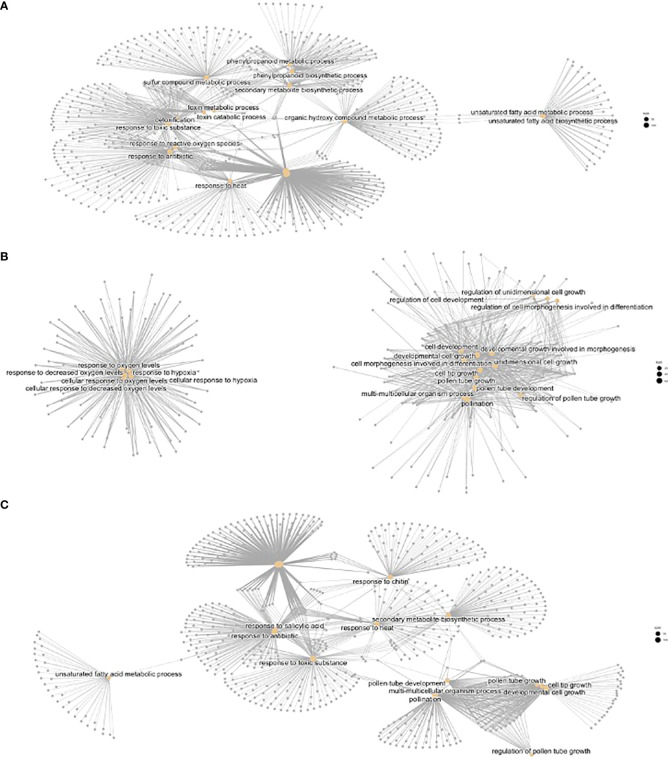
CNET plots of Biological Functions with differential regulation. **(A)** Heat vs control, **(B)** cold vs control, **(C)** heat vs cold.

**Figure 6:**
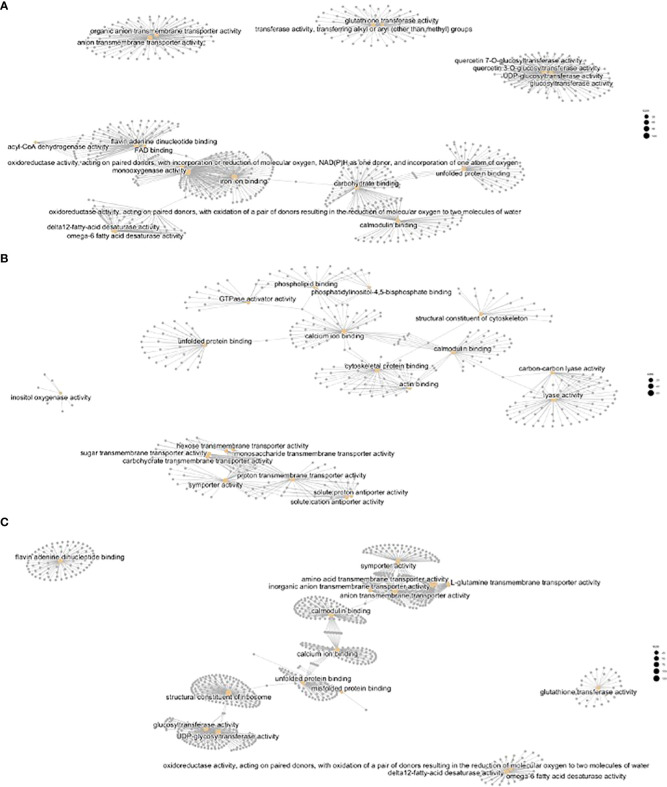
CNET plots of Molecular Functions with differential regulation. **(A)** Heat vs control, **(B)** cold vs control, **(C)** heat vs cold.

Protein synthesis is affected by cold in many plants (Cheong et al., 2021), and indeed we found a large dataset of 284 genes coding for ribosomal components that were differentially affected by cold (**Figures 5**, **Figure 6**). These included genes coding for ribosomal proteins S2, S3, S4, S9, L7, L11, L24, L27, or L32. The major changes observed in ribosomal proteins were also accompanied by changes in gene expression of genes involved in ribosomal biogenesis, such as Am01g11680, orthologous to *SMOG4*. This homolog of yeast NOP53 is involved in maturation of 5.8rRNA, and has a strong effect in cell proliferation in Arabidopsis (Micol-Ponce et al., 2020). The down regulation of the genes coding for ribosomal genes appears to affect most paralogs present in the genome. Indeed, there were four paralogs of the S4/S9 terminal domain, three out of four S12/S23, and three out of four S7p/S5e paralogs were down regulated, suggesting a major reprogramming of the ribosomal structure.

We found that the molecular function (MF) enriched for unsaturated fatty acid metabolism comprised 33 fatty acid desaturases, three SnrK2-like proteins, and 13 fatty acid desaturases involved in changes of fatty acids in the endoplasmic reticulum. Changes in the membrane’s biophysical properties are associated to modified fatty acid composition, indicating an adaptation to differing temperatures (see discussion). Together with changes in unsaturated fatty acid metabolism, we found MF related to transport of carboxylic acids, amino acids, inorganic anions, and glutamine.

The transcriptional structure found in long-term cold and hot growing conditions, thus showed a large set of enriched fractions corresponding to the different phenotypes identified, such as pollen growth, anthocyanin metabolism, or VOC synthesis. This apparent coincidence is only partial, as we sampled petals, not pollen (see discussion). In addition, we found hypoxia and a major reprograming of the ribosomal machinery. Our results indicate that a long-term exposure to non-optimal temperatures has a different transcriptional signature, compared to the classic short-term heat/cold shocks.

### 3.6 Response of developmental and signaling processes to temperature changes

While GEA/CNET identifies major changes in biological pathways, some genes involved in development and signaling do not appear in this type of analysis. Phytochrome B is a major gene involved in both light and temperature signaling (Legris et al., 2016). The *Antirrhinum* ortholog of PHYB Am01g64810.T01, was differentially expressed between cold and heat. However, PIF4 (Am02g50260) did not significantly changed in expression. It is involved in the activation of auxin synthesis and cell expansion at high temperature in Arabidopsis (Franklin et al., 2011) and we did not find cell expansion changes. The ortholog of FT (Am01g05660), was not differentially expressed. However, a phosphatidyl glycerol phosphatase 1, controlling FT movement (Susila et al., 2021), and its activator CO (Am03g06610), were differentially expressed, indicating that the apparent differences in flowering time observed in the different temperature treatments (unpublished results) were caused *via* the conserved FT-CO signaling pathway.

Temperature had a stronger effect on perianth organs, i.e. sepals and petals, than in stamens and carpels. We found that both B function genes *DEFICIENS* (Am01g03890) and *GLOBOSA* (Am04g30510) were differentially expressed, together with *SQUAMOSA* and *DEFH200* (Am06g13650). These results support the phenotypes found after treatment with non-optimal temperatures, as genes involved in perianth development were affected.

The so-called temperature compensation is thought to be a mechanism where plants modify their metabolism to maintain an internal pace irrespective of temperature. Circadian clock genes are involved in temperature compensation (Gould et al., 2006). Although our transcriptomic sampling was not performed in a circadian fashion, we observed significant changes in expression of the core clock genes *AmLHY* and *AmTOC1*. The genes coding for the evening complex *AmELF3* and *AmGIGANTEA*, involved in stabilization of ZTL (Kim et al., 2007), were also differentially expressed in cold versus heat, and cold versus control, suggesting a modified circadian transcriptome resulting from long-term temperature changes.

## 4 Discussion

In this work, we analyzed the long-term effect of non-optimal growing temperatures on flower development of *Antirrhinum majus*. We chose two temperatures ranges that may correspond to a cold spring with temperatures of 15°/5° C degrees day and night, and a mild summer with temperatures of 30°/23°C. While both temperature regimes are not extreme, they had a very strong impact on growth, as compared to standard (control) 22°/16°C day/night temperatures. This effect should be reflected in steady state transcriptomics after long periods of non-optimal temperatures.

We found that flower development was affected in almost every aspect, although some areas of robustness were identified. Floral organ development occurs from floral primordia, and just like leaves, it is comprised of an initial growth process driven by cell division, and a second part where cell expansion takes over (Reale et al., 2002; Delgado-Benarroch et al., 2009a; Gonzalez et al., 2012). Environmental changes, such as low or high temperatures, affect cell division and expansion (Granier et al., 2000). The most noticeable changes in floral organ size were observed in sepals and petals. Our transcriptomic analysis showed that the major genes involved in petal development i.e. *DEF* and *GLO*, were significantly affected by temperature. The only floral organ that remained unchanged was the petal tube. A number of studies have previously shown that the petal tube and the petal limb may be considered different in terms of regulatory networks related to growth or even pigment accumulation (Wheldale and Bateson, 1907; Almeida et al., 1989; Manchado-Rojo et al., 2014). Our work shows that the significant differences found in petal size under different temperature growth regimes were mainly due to changes in cell division. Similar results have been obtained under shorter growth periods in heat in Petunia (Sood et al., 2021). There are two mechanisms that render flowers with a lower or higher total cell number that are not mutually exclusive. The first is a change in the size of the flower meristem, and the second is a change in the period or speed of petal cell division. As our results showed that temperature only affected cell division, a differing floral size may be the result from changes in meristem size and petal growth (see below).

As other tissues in which meiosis occurs, pollen production is highly sensitive to temperatures. There is a large body of works that describe the effects of non-optimal temperatures on pollen development, including *Antirrhinum* species (Diers, 1971; Alaimo et al., 1997). Non-optimal temperatures affect pollen development in many plants. These include cold temperature in soja and tomato (Fernandez-Munoz et al., 1995; Domínguez et al., 2005; Ohnishi et al., 2010). High temperatures cause male sterility in rice (Endo et al., 2009), and again anther and pollen developmental defects in tomato (Müller et al., 2016). The defects in anther and pollen development in tomato were traced back to the B function genes that we found to be down-regulated (see below). Thus, it did not come as a surprise that high temperatures caused decreased pollen viability. Sets of enriched genes related to pollen and reproduction were identified despite using petals as a tissue. This indicates that the genes that are down regulated in petals, may be related, not only to pollen germination, growth, and viability, but may also play additional roles in other organs. Concerning seed germination, early studies have shown that temperatures play a key role in the process (Morinaga, 1926). Seed germination and viability is the result of developmental processes affected by environmental conditions. Abiotic stresses such as salt, cold and water stress negatively affect seed filling and impact seed germination in rice (Schmidt et al., 2013; Liu et al., 2019). As we found improved pollen germination at low temperatures. However, seed germination and quantity are optimal under control conditions. We conclude that fertilization and seed filling play key roles in *Antirrhinum* seed viability. Probably each of these processes are unequally affected by temperature but the optimal combination of pollen germination, fertilization and seed filling occurs at temperate temperatures.

The existence of both petal color and scent as cues for pollinators have been studied in many systems. Both color and scent are important for pollinator attraction in the genus *Anthirrinum* and other species (Hoballah et al., 2007; Suchet et al., 2010; Amrad et al., 2016; Ruiz-Hernández et al., 2021). Our results show an interesting coordination of both traits, as cold-grown plants showed a stronger and brighter color, while heat-grown plants were paler but had a richer scent profile, as compared to cold-grown flowers. Low temperatures activate anthocyanin biosynthesis while high temperatures have the opposite effect in apple fruits (Lin-Wang et al., 2011; Bai et al., 2014). Furthermore, the accumulation of anthocyanins in red oranges is controlled *via* a temperature sensitive retrotransposon (Butelli et al., 2012). Under cold conditions, having a bright color might be advantageous for pollinator attraction. Studies in Antirrhinum have shown the importance of anthocyanins for pollinator attraction (Dyer et al., 2007).

Volatile emission is affected by temperatures in many plants. Indeed, high temperatures modify scent profiles and emission in Petunia (Sagae et al., 2008; Cna’ani et al., 2015). Work in *Jasminun* has shown a maximum of emission at 25° with significantly lower levels at 20° or 30°C (Barman and Mitra, 2021).Similar to the results found for *Antirrhinum* flowers, we found in a previous work that the scent complexity of Narcissus cut flowers decreases when stored at cold temperatures and increased at higher temperatures (Terry et al., 2021). This indicates that the multicomponent functionality of flowers to attract pollinators may be advantageous given the climatic cycles observed in nature.

Several BP and MF categories appeared as a response to long-term temperature changes indicating basal adaptation. One was a major reorganization of ribosomal protein paralogs comprising over 200 genes. The ribosome acts as a cryosensor in plants, as reducing ribosomal activity induces cold signaling *via* CBF (Wigge et al., 2020). Changes in ribosome structure in response to short-period temperature changes have been recorded in *E.coli*, zebrafish and a variety of plants (VanBogelen and Neidhardt, 1990; Long et al., 2013; Martinez-Seidel et al., 2021). The number of paralogs coding for ribosomal proteins appears to be between two and over 30 in plants (Martinez-Seidel et al., 2020). Previous work describing modified ribosomal compositions coined the concept of ribosomal heterogeneity (Horiguchi et al., 2012). It appears that different ribosomal protein paralogs are not functionally redundant, as mutations in single genes cause severe developmental phenotypes such as altered leaf polarity or embryo lethality (Ma and Dooner, 2004; Fujikura et al., 2009). Recent work has shown that single amino acid changes in the ribosomal protein RPS23 have a strong impact on protein quality and aging in yeast, worms and flies, making them heat resistant (Martinez-Miguel et al., 2021). The *Antirrhinum* ortholog of RPS23 (Am01g28590) was differentially expressed in the dataset. This indicates that overall environmental impact may be channeled *via* changes in the protein synthesis machinery. These ribosomal types may be part of a basal adaptation to non-optimal temperatures.

Cellular membranes play a key role in every aspect of a cell’s functions, including signaling and transport. The analysis shows that unsaturated fatty acid biosynthesis may be part of long-term adaptation to modified temperatures. The genes found indicate an adaptation of plasma and endoplasmic membranes. Adaptation to cold at the membrane level occurs in bacteria (Sakamoto and Murata, 2002), and functional studies have shown a positive effect of desaturation in tobacco (Kodama et al., 1994). This indicates that long-term cold stress may cause a reprograming of cell membranes as an adaptive step. Indeed, one group of enriched genes was identified as transporters of amino acids, cations etc. As these proteins are membrane bound, an emerging hypothesis is that there is a major reprogramming of both membrane structure and transporters. However, we do not have data to support changes at the paralog level versus decreased/increased activity of some transporters that may become limiting for growth and development.

Hypoxia has been extensively studied because of water logging in roots (Drew, 1997). The response to hypoxia in flooded roots occurs *via* a signal transduction pathway coordinated by type VII ETHYLENE RESPONSE FACTORS and the ABA signaling pathway (González-Guzmán et al., 2021). However, we sampled petals, a tissue with only three cell layers. We hypothesize that petals may be especially sensitive to temperature, as they are non-photosynthetic organs. This may compromise the equilibrium between sugar intake, photorespiration, and metabolism, making them vulnerable, and becoming hypoxic, despite their vicinity to the atmosphere. Hypoxia causes changes in protein degradation (Gibbs et al., 2011). Thus, the phenotypes observed may be the result of modified cellular processes that include protein synthesis and degradation. Due to the lengthy period required to flower in the cold, and the reduced number of flowers produced under heat conditions, we did not quantify floral number. However, considering the importance of the hypoxia niche in the shoot apical meristem (Weits et al., 2019), it could be that the observed changes in growth could be partly due to a modified state of hypoxia. This may cause changes in the speed of lateral organ formation, resulting in modified speeds of flower formation and changes in flower size due to differing size in primordia.

The hypoxia molecular footprint was different from that described for roots in other species such as tomato (Safavi-Rizi et al., 2020) or Arabidopsis (Gasch et al., 2016; Tang et al., 2021). Indeed, we found several AP2 transcription factors such as RAP2.2, DREB2 and DREB2A that respond to low oxygen levels. The ADH gene is a direct target of RAP2.2 in Arabidopsis in response to low oxygen levels (Papdi et al., 2015). As *AmADH1* was differentially expressed we infer that despite the sampling of petals, there is a hypoxic niche in this tissue occurring because of non-optimal temperatures. Although ABA is an inherent part of the hypoxia signaling response (González-Guzmán et al., 2021), only one paralog of PYL4 *Am07g22130*, showed differential expression indicating that the hypoxia signaling may vary between tissues. Indeed, hypoxia plays a role in root architecture *via* modification of the auxin signaling (Shukla et al., 2019). We did not find differential expression of auxin signaling genes. As petals do not undergo further growth, we conclude that the effects of hypoxia on auxin signaling could have occurred early in petal development but are absent at late stages of development.

While the GE analysis showed major pathways, small pathways with a few genes or unannotated components are difficult to find. We found two major players in flower development i.e. floral organ identity and circadian clock genes, and both showed differential expressions. Floral organ identity genes encoding the B -function involved in petal development have a major impact in floral organ size in *Antirrhinum* and petunia (Bey et al., 2004; Rijpkema et al., 2006). We indeed found differential expressions of DEF and GLO, involved in promoting flower size and scent emission (Manchado-Rojo et al., 2012). The plant’s circadian clock coordinates cell division (Fung-Uceda et al., 2018), and *AmLHY* is a positive regulator of flower development (Terry et al., 2019). Furthermore, flower scent is misregulated in knockdown lines of *Antirrhinum* and petunia ZTL/CHL, GI1, or LHY (Fenske et al., 2015; Terry et al., 2019; Brandoli et al., 2020). While our sampling was at a single point, the deregulation of clock genes suggests that the identified changes in scent emission may be the result of a loss of both floral organ identity and clock gene coordination.

## 5 Conclusions

Exposure to short term heat or cold temperatures causes changes in gene expression, and our work showed that some of these processes, such as protein folding related to heat, ribosomal structure, or membrane fatty acid composition, are inherent to the process of adaptation. We propose that climate change has two levels of action in plants, one *via* the adaptation of basic cellular functions, and a second one related to tissue or development-specific processes. Differing genetic capacities in launching these processes may underlie the observed differences in resilience. Furthermore, not all biological processes may be equally affected by non-optimal temperatures. While *Antirrhinum majus* is an ornamental crop, the effects of non-optimal temperatures in flower development are profound. They affect all aspects including seed production. This indicates that obtaining resilient flowers is part of the requirements to provide food security. The effects on scent emission and flower color open another front related to pollination, with both ecological and economical implications for many crops.

## Data availability statement

The data presented in the study are deposited in the European Nucleotide Archive (ENA) https://www.ebi.ac.uk/ena/browser/home (Study: PRJEB54068, Samples: ERS12336218-ERS12336232, Experiments: ERX9450224-ERX9450238, Runs: ERR9907396-ERR9907410). The description of this additional annotation process and the resulting files are available at https://github.com/jesualdotomasfernandezbreis/snapdragon-annotation.

## Author contributions

RA: Conceptualization, methodology, investigation, data curation. JW: Conceptualization, methodology, investigation, validation, formal analysis, resources, data curation, writing, supervision, funding acquisition, project management. MT: Formal analysis, data Curation. NB: Formal analysis, data Curation. FV-N: Formal analysis, data curation. JF-B: Conceptualization, methodology, writing - review & editing, project management. ME-C: Conceptualization, methodology, investigation, validation, formal analysis, resources, data curation, writing, supervision, funding acquisition, project management. All authors contributed to the article and approved the submitted version.
