# Supplementary material for: Flower transcriptional response to long term hot and cold environments in Antirrhinum majus
Source: Front Plant Sci. 2023 Jan 27;14:1120183. doi: 10.3389/fpls.2023.1120183 (PMC9911551; doi:10.3389/fpls.2023.1120183)

Supplementary Material

Flower transcriptional response to long term Hot and Cold environments

Raquel Alcantud^1^, Julia Weiss^1^, Marta I. Terry^1^, Nuria Bernabé^3^, Fuensanta Verdú-Navarro^1,2^, Jesualdo Tomás Fernández-Breis^3^, Marcos Egea-Cortines^1^

*** Correspondence:** [marcos.egea@upct.es](mailto:marcos.egea@upct.es) : marcos.egea@upct.es

# Supplementary Figures and Tables

**Supporting Information**, **Table S1**. Statistical analysis of snapdragon floral parameters. Multiple comparisons were calculated by Wilcoxon Test. A *p* value < 0.05 denotes a significant difference. P1: petal tube length; P2: lower petal length; P3: petal height; P4: sepal length; P5: tube width; P6: upper petal length; P7: lower petal expansion; P8: upper petal expansion; P9: stamen length; P10: gynoecium length; P11: pallate expansion.

| **Parameter** | **Group** | **Heat** | **Cold** |
| --- | --- | --- | --- |
| P1- petal tube length | Cold | 1 | - |
|  | Control | 1 | 1 |
| P2- lower petal length | Cold | 0.0021 | - |
|  | Control | 0.0015 | 0.7959 |
| P3- petal height | Cold | 0.0000 | - |
|  | Control | 0.0000 | 0.2176 |
| P4- sepal length | Cold | 0.0091 | - |
|  | Control | 0.0051 | 0.3930 |
| P5- tube width | Cold | 0.0104 | - |
|  | Control | 0.0045 | 0.3150 |
| P6- upper petal length | Cold | 0.0005 | - |
|  | Control | 0.0001 | 0.0639 |
| P7- Lower petal expansion | Cold | 0.0001 | - |
|  | Control | 0.0433 | 0.0104 |
| P8- upper petal expansion | Cold | 0.0013 | - |
|  | Control | 0.0147 | 0.0034 |
| P9- stamen length | Cold | 0.0001 | - |
|  | Control | 0.0001 | 0.6842 |
| P10- gynoecium length | Cold | 0.0006 | - |
|  | Control | 0.0002 | 0.0288 |
| P11- pallate expansion | Cold | 0.0006 | - |
|  | Control | 0.0006 | 0.6305 |

**Supporting Information, Table S2**. Pedicel length statistical analysis, Differences between different temperature groups were tested with Wilcoxon’s Test. A *p* value < 0.05 denotes a significant difference.

| **Group** | **Heat** | **Cold** |
| --- | --- | --- |
| Cold | 0.0000 | - |
| Control | 0.0000 | 0.5488 |

**Supporting Information, Table S3**. Statistical analysis of anthocyanin between snapdragon groups by using Wilcoxon’s Test. A *p* value < 0.05 denotes a significant difference.

| **Group** | **Control** | **Heat** |
| --- | --- | --- |
| Heat | 0.01061 | - |
| Cold | 0.00012 | 0.00012 |

**Supporting Information, Table S4**. Statistics results of chlorophyll content differences from different leaves (apical, median and basal position), between different temperature conditions. The analysis was performed with Wilcoxon’s Test. A *p* value < 0.05 denotes a significant difference.

| **Leaf** | **Group** | **Heat** | **Cold** |
| --- | --- | --- | --- |
| Apical | Cold | 0.023 | - |
|  | Control | 0.845 | 0.024 |
| Median | Cold | 0.002 | - |
|  | Control | 0.878 | 0.002 |
| Basal | Cold | 0.040 | - |
|  | Control | 0.426 | 0.009 |

**Supporting Information, Table S5**. Statistical comparison of total scent emission at different developmental stages. For every temperature group, total volatile concentrations were compared between each day after anthesis (DAA) combinations. A *p*-value < 0.05 indicates a significant difference.

| **Group (temperature)** | **DAA** |  | ***p*-value** |
| --- | --- | --- | --- |
| Control | 0 | 3 | 0.076 |
|  | 0 | 5 | 0.008 |
|  | 3 | 5 | 0.270 |
| Heat | 0 | 3 | 0.198 |
|  | 0 | 5 | 0.458 |
|  | 3 | 5 | 0.416 |
| Cold | 0 | 3 | 0.750 |
|  | 0 | 5 | 0.687 |
|  | 3 | 5 | 0.867 |

**Supporting Information, Table S6.** Total scent emission multiple comparison between temperatures. For every analyzed floral stage (days after anthesis, DAA), the total amount was compared between each temperature condition combinations. A *p*-value < 0.05 indicates a significant difference.

| **DAA** | **Group (temperature)** | | ***p*-value** |
| --- | --- | --- | --- |
| 0 | Control | Heat | 0.524 |
|  | Control | Cold | 0.504 |
|  | Heat | Cold | 0.686 |
| 3 | Control | Heat | 0.046 |
|  | Control | Cold | 0.01 |
|  | Heat | Cold | 0.436 |
| 5 | Control | Heat | 0.007 |
|  | Control | Cold | 0.001 |
|  | Heat | Cold | 0.461 |


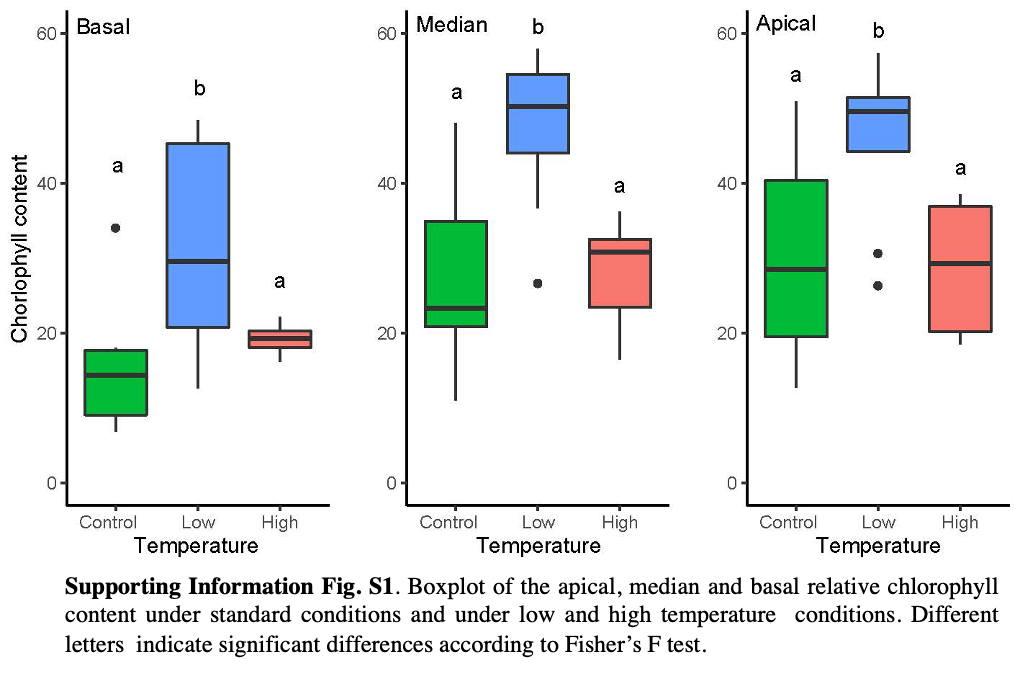


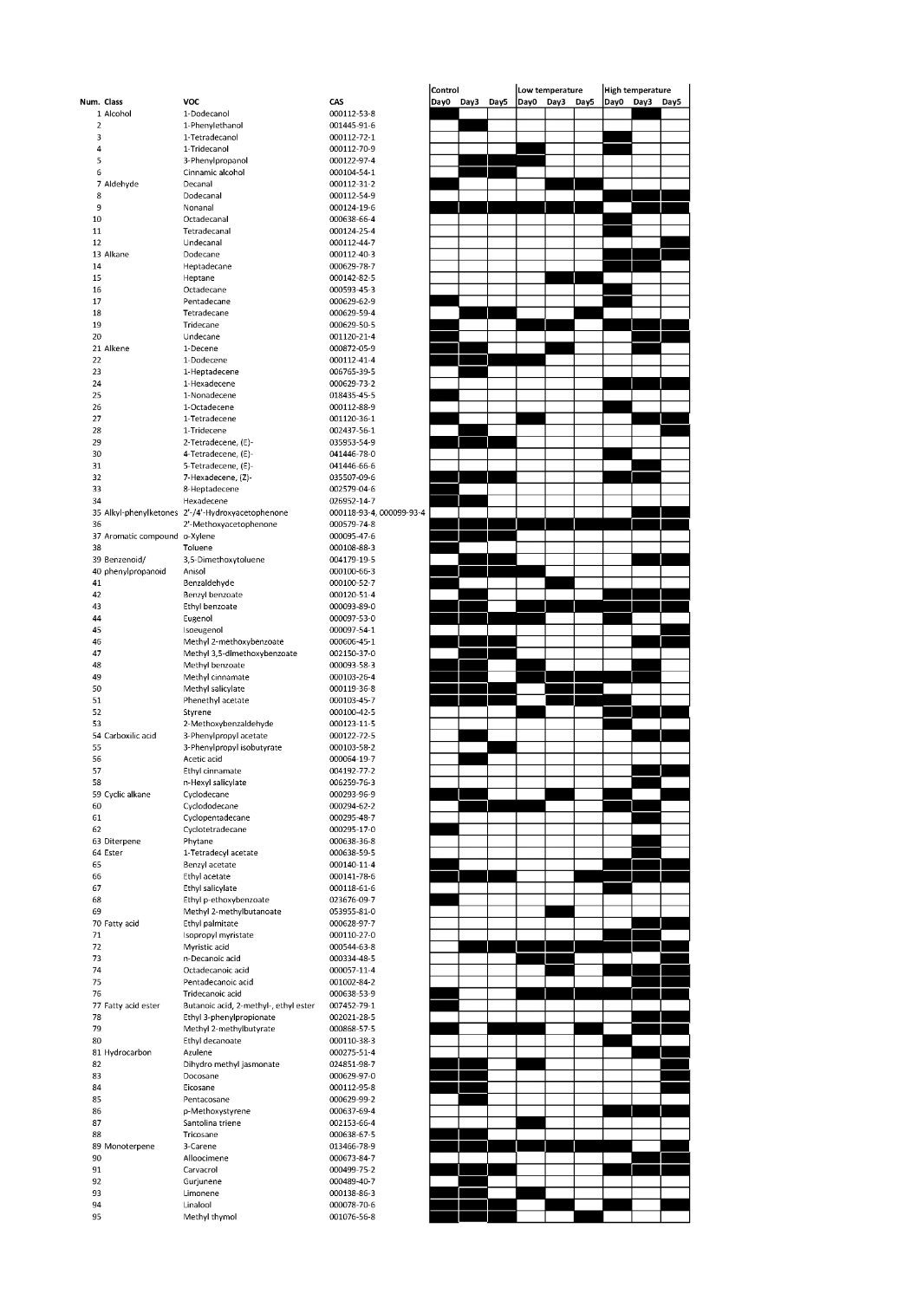


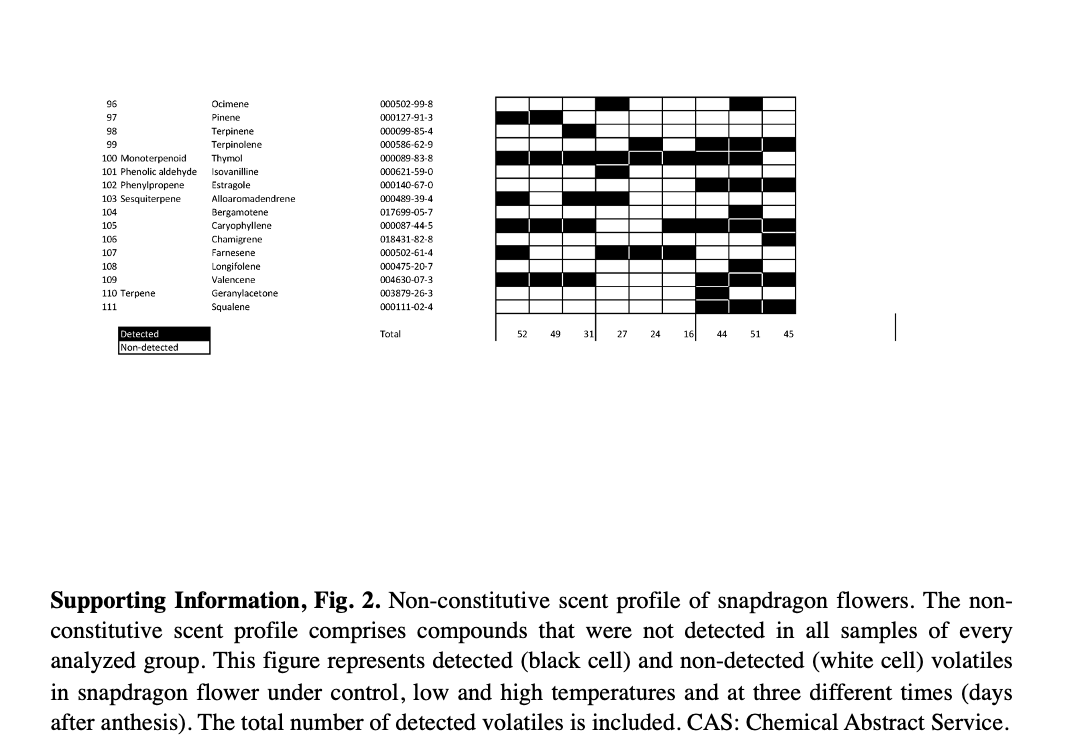


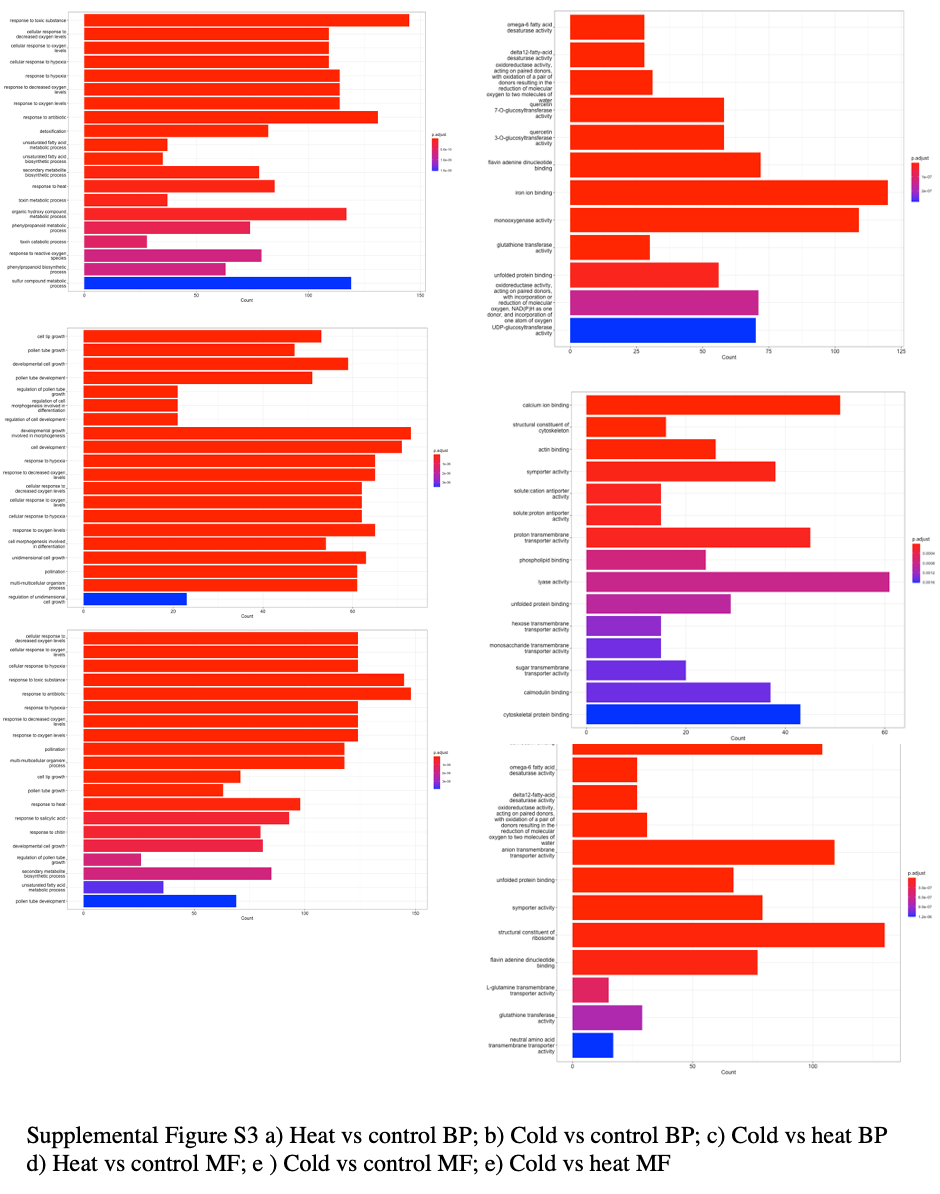

Supplement: Supplementary file 1 [file DataSheet_1.docx]
